# 

**Published:** 1895-10

**Authors:** 


### CONTENTS FOR OCTOBER, 1895.

| ORIGINAL COMMUNICATIONS. | PAGE. |
|---|---|
| Bruises of the Lungs. By JOHN PARMENTER, M. D | 209 |
| Alcoholism with Suggestions as to Treatment—Statistics for Buffalo. By SIDNEY A. DUNHAM, M. D | 217 |
| The Value of Antiseptics in Surgery. By WILLIAM STANTON, M. D | 224 |
| A New Method of Stomach Washing. By JOHN T. PITKIN, M. D | 228 |
| Flooding the Urinary Tract. By B. H. DAGGETT, M. D | 231 |
| **PROGRESS IN MEDICAL SCIENCE.** | |
| Neurology | 241 |
| Obstetrics, Gynecology and Pelvic Surgery | 246 |
| Public Health, Hygiene and Bacteriology | 258 |
| An Important Point in the Examination of Sputum | 260 |
| **CORRESPONDENCE.** | |
| Berlin Letter | 261 |
| **EDITORIAL.** | |
| The Noise Nuisance Again | 265 |
| Public Baths | 269 |
| Topics of the Month | 269 |
| Personal | 276 |
| Society Meetings | 277 |
| Reviews | 279 |
| Miscellany | 288 |

